# Work-related diabetes distress among Finnish workers with type 1 diabetes: a national cross-sectional survey

**DOI:** 10.1186/s12995-016-0099-4

**Published:** 2016-03-21

**Authors:** Pirjo Hakkarainen, Leena Moilanen, Vilma Hänninen, Jarmo Heikkinen, Kimmo Räsänen

**Affiliations:** School of Medicine, Institute of Public Health and Clinical Nutrition, University of Eastern Finland, Kuopio, Finland; Department of Medicine, Kuopio University Hospital, Kuopio, Finland; Department of Social Sciences, University of Eastern Finland, Kuopio, Finland

**Keywords:** Type 1 diabetes, Work, Stress, Exhaustion, Blood glucose

## Abstract

**Background:**

Diabetes distress is common among people with type 1 diabetes, but knowledge is scarce regarding the perceived burden of reconciling work with this disease. This cross-sectional study investigated work-related diabetes distress among Finnish workers with type 1 diabetes.

**Methods:**

A questionnaire was mailed to 2500 randomly sampled 18- to 65-year-old Finns with type 1 diabetes; 49.3 % responded. Work-related diabetes distress was measured by combining worry and exhaustion in reconciling work with diabetes. Self-perceived work-related diabetes distress was evaluated in the context of physical and psychosocial work conditions, job demands, work ability, general stress, diabetes acceptance, glycosylated hemoglobin (HbA1c) level, high blood glucose maintenance at work, and depressive symptoms. The data were analyzed with the use of cross-tabulation, chi-square tests, ANOVA analysis, Spearman correlation coefficients, and structural equation modeling.

**Results:**

Of the respondents, 70 % experienced work-related diabetes distress. Problems with physical work conditions (β = 0.27), work ability (β = -0.21), difficulty in accepting diabetes (β = 0.18), and job demands (β = 0.14) were found to be associated with work-related diabetes distress. This distress was strongly associated with the maintenance of a high blood glucose level at work (β = 0.34). In turn, a high blood glucose level at work was associated with a high HbA1c level (β = 0.29). Work-related diabetes distress and depressive symptoms had a bi-directional association (β = 0.06 and β = 0.14). Difficulty accepting diabetes had three-dimensional associations: work-related diabetes distress (β = 0.18), depressive symptoms (β = 0.13), and high HbA1c level (β = 0.12). There was no notable association between work-related diabetes distress and general stress.

**Conclusions:**

Work-related diabetes distress is common among workers with type 1 diabetes, and it may influence metabolic control. This stress could be prevented by adapting physical work conditions. People with type 1 diabetes should also be encouraged to pursue their full educational potential, and psychological support should be provided for those with difficulty accepting their diabetes.

**Electronic supplementary material:**

The online version of this article (doi:10.1186/s12995-016-0099-4) contains supplementary material, which is available to authorized users.

## Background

Diabetes distress is common among people with type 1 diabetes [1]. This psychological stress is related to living with diabetes and involves an emotional burden, physician-related and regimen-related stress, and diabetes-related interpersonal stress (i.e., lack of social support) [1–3]. Diabetes distress is strongly associated with poor diabetes self-management, and it is more common than depression among people with diabetes [3, 4].

Harmful work stress occurs when job demands exceed people’s resources for handling their jobs [5, 6]. It is a significant predictor of anxiety and depression [7–10]. In addition, self-reported exhaustion has been found to be associated with anxiety, depression, burnout, and poor work ability [11].

The number of persons with type 1 diabetes is increasing in the workforce, but little is known about the relationship between work and type 1 diabetes [12, 13]. Previous studies have primarily focused on work productivity [14–16] and work disability [15, 17] among people with diabetes, but knowledge about the perceived burden of reconciling work with type 1 diabetes is scarce [18].

The aim of this cross-sectional study was to examine work-related diabetes distress among Finnish workers with type 1 diabetes. Self-perceived work-related diabetes distress was evaluated in the context of work conditions, job demands, work ability, general stress, diabetes acceptance, glycemic control, and depressive symptoms.

## Methods

This study was carried out as part of a project called “People with Type 1 Diabetes in Worklife”, conducted by the University of Eastern Finland and the Kuopio University Hospital in 2010–2012. The Research Ethics Committee of the Northern Savo Hospital District reviewed and approved the research protocol (18//2010).

The study questionnaire was constructed on the basis of previously validated worklife scales and diabetes questionnaires [19–24]. The questionnaire was mailed to a random sample of 2500 Finns with type 1 diabetes between the ages of 18 and 65 years and drawn from The Medication Reimbursement Register of The Social Insurance Institution of Finland, which includes all Finns with type 1 diabetes. Non-responders were sent two reminders. There were slightly over 40 000 persons with diagnosed type 1 diabetes in Finland in 2011 [25]. Thus, this random sample covered 6 % of the Finnish population with type 1 diabetes.

Altogether 2464 persons received the questionnaire (4 deceased and 32 unreachable), and 1214 returned the form. Thus the response rate was 49.3 %. We received 126 forms from respondents who reported having been diagnosed with another type of diabetes, and they were rejected. In addition, 25 respondents had been diagnosed after retirement, 16 were incapable of responding on account of a disability or serious illness, 15 declared that they were not working (in the last 12 months), 6 were not able to fill out the form in Finnish, and 13 had other reasons for not responding. These 75 uncompleted forms were also excluded from the study. Therefore the final sample included 1013 persons with type 1 diabetes.

There was a small difference in the gender distribution between the original sample and the respondents (proportion of men being 65.3 % in the original and 57.5 % among the respondents). The distributions of the age groups and residential provinces were similar in both groups (dispersion about 1 %).

We studied Finnish workers with type 1 diabetes. Of the total sample of 1013, those who were retired, unemployed, students, homemakers, and others who did not participate in worklife during the past 12 months were excluded, leaving 767 respondents to be included in the analysis.

### Measurements

#### Work-related diabetes distress

On a scale of 1–3 (never; sometimes; often), the respondents rated how often they worried about their ability to do their job due to their diabetes. Using the same scale, they also rated how often they became exhausted by the need to reconcile their work with their diabetes. These variables correlated strongly (Spearman’s correlation coefficient 0.64, *P* < 0.001). Thus the sum index “work-related diabetes distress” was calculated on the basis of these variables. In previous studies diabetes distress has been found to predict poor diabetes self-management and poor glycemic control [1, 26]. Diabetes-specific stress can also increase the risk of depression [27]. In addition, it has been found that diabetes distress mediates the relationship between depression and glycemic control [28].

#### Sociodemographic variables

The sociodemographic variables included gender, age, marital status, duration of diabetes, educational level, type of employment, and type of work.

#### Work-related variables

##### Problems with physical work conditions

The respondents were asked: “Do problems in the work environment or with the physical load of work hinder you managing or coping with your work?” [19]. The options (1–4) were not present/no harm; troubles a little; troubles a lot; cannot say. For our statistical analysis, those who reported option 4 = cannot say (*n* = 13) were combined with option 1 = not present/not harm. In a previous study, it was found that people who had a high physical load at work perceived diabetes self-management as a burden [29].

##### Problems with psychosocial work conditions

The respondents were asked: “Do problems in the work community or the mental load of work hinder you managing or coping with your work?” [19]. The options (1–4) were not present/no harm; troubles a little; troubles a lot; cannot say. For our statistical analysis, those who reported option 4 = cannot say (*n* = 15) were combined with option 1 = not present/no harm. Problems with psychosocial work conditions have been found to predict mental distress and depression [30, 31].

##### Job demands

Five questions of the Job Content Questionnaire [20] that have been widely used to assess job demands [32] and job strain [33] were included in the questionnaire. The questions were (a) “My job requires me to work very fast”, (b) “My job requires me to work very hard”, (c) “My job requires an inordinate amount of work”, (d) “My job requires an intense pace”, (e) “I have enough time to get my job done.” For our further analyses, the scale of question e was reversed. The respondents rated these features of their jobs on a scale of 1–5 (fully agree to fully disagree). From these items, a sum index was calculated. Job demands have been found to form a risk factor for perceived stress and psychological distress [34–36].

#### Health-related variables

##### Depressive symptoms

To assess current depressive symptoms or a high risk of depression, we asked the respondents to answer “yes” or “no” to the following two questions: “During the past month, have you often felt down, depressed, or hopeless?” and “During the past month, have you often felt little interest or pleasure in doing things?”. A sum index was calculated. These two questions have been developed for screening purposes and have proven to be sensitive and specific for the screening of depression [21]. A bi-directional relation has been found between diabetes distress and depressive symptoms: diabetes distress predicting depressive symptoms and depressive symptoms amplifying diabetes distress [37]. Depression can also cause poor diabetes self-care [38].

##### Work ability

Work ability was measured with the use of the Work Ability Score (WAS), which measures self-reported work ability by comparing a person’s current work ability with the person’s lifetime best on a scale of 0–10 (0 = completely unable to work to 10 = work ability as its best) [22, 39]. This single-item question is the first item of the widely used Work Ability Index, WAI [40]. Both, WAS and WAI have been found to predict stress, general and mental health, and sick leaves [22].

##### General stress

General stress was asked by the question: “Do you feel stress these days?” [23]. The respondents rated their perceived stress on a scale of 0–4 (not at all – very much). In previous studies stress has generally been found to predict anxiety and depression [9].

#### Diabetes-related variables

##### Glycosylated hemoglobin level

The respondents were asked to report their last glycosylated hemoglobin (HbA1c) level using the categories ≤60 mmol/mol (≤7.5 %), 61–70 mmol/mol (7.6–8.5 %), 71–80 mmol/mol (8.6–9.5 %), and ≥81 mmol/mol (≥9.6 %) [24].

##### High blood glucose level at work

On a scale of 1–5 (never to always), the respondents rated how often they kept their blood glucose level higher at work than usual.

##### Difficulty in accepting diabetes

On a scale of 0–2 (no; yes, a little; yes, a lot), the respondents were asked: “Do you have difficulty in accepting your diabetes?” Difficulty in accepting diabetes has been found to be associated with reduced self-care, a high HbA1c level, diabetes distress, and depressive symptoms [41].

##### Severe hypoglycemia events in the past 12 months

On a scale of 0–3 (no; once; 2–3 times; more often), the respondents were asked: “Have you had hypoglycemic events in which help has been needed in the last 12 months?”.

### Statistical analysis

The number of missing values varied between 0 and 22 among the studied variables. Missing values were substituted by the mean (ratio scaled factors) or mode (nominal and ordinal scaled factors).

For further analysis, we dichotomized the nominal scaled variables gender, marital status, type of employment, and type of work. Educational level, physical work conditions, psychosocial work conditions, general stress, HbA1c level, high blood glucose level at work, difficulty in accepting diabetes, and severe hypoglycemic events were ordinal scaled, and they were treated as continuous variables. We computed age and duration of diabetes as original continuous scores and calculated the values for work-related diabetes distress, job demands, depressive symptoms, and self-rated work ability as additive sums.

The distributions of the relevant variables in the sample were analyzed in association with work-related diabetes distress by cross-tabulation, the chi-square test, and an ANOVA analysis (Table 1). Spearman’s correlations were calculated between the background and independent and dependent variables (Table 2).

**Table 1:** Characteristics of the respondents according to their work-related diabetes distress

	All *N* = 767	Never stressed *n* = 230 (30.0 %)	Sometimes stressed *n* = 379 (49.4 %)	Often stressed *n* = 158 (20.6 %)	*P*-value
Gender n (%)					<0.001^a^
Male	430 (56.1)	150 (34.9)	213 (49.5)	67 (15.6)	
Female	337 (43.9)	80 (23.7)	166 (49.3)	91 (27.0)	
Age, years (mean ± SD)	36.2 ± 12.4	34.4±12.9	36.3±11.8	38.7±12.6	0.004^b^
Marital status, n (%)					0.895^a^
Married or cohabiting	478 (62.3)	142 (29.7)	235 (49.2)	101 (21.1)	
Unmarried, divorced, widowed	289 (37.7)	88 (30.4)	144 (49.8)	57 (19.7)	
Duration of diabetes, years (mean ± SD)	8.5 ± 4.8	9.1±4.7	8.1±4.8	8.6±4.7	0.050^b^
Educational level, n (%)					0.003^a^
Basic education	182 (23.7)	60 (33.0)	78 (42.9)	44 (24.2)	
Vocational school	264 (34.4)	68 (25.8)	142 (53.8)	54 (20.5)	
Technical or vocational college	217 (28.3)	56 (25.8)	112 (51.6)	49 (22.6)	
University or university of applied science	104 (13.6)	46 (44.2)	47 (45.2)	11 (10.6)	
Employment, n (%)					0.318^a^
Entrepreneur	68 (8.9)	16 (23.5)	34 (50.0)	18 (26.5)	
Employee	699 (91.1)	214 (30.6)	345 (49.4)	140 (20.0)	
Type of work, n (%)					0.016^a^
Mental work	324 (42.2)	115 (35.5)	150 (46.3)	59 (18.2)	
Physical work	443 (57.8)	115 (26.0)	229 (51.7)	99 (22.3)	
Problems with physical work conditions, n (%)					<0.001^a^
Not present/no harm/cannot say	469 (61.1)	198 (42.2)	220 (46.9)	51 (10.9)	
Troubles a little	248 (32.3)	29 (11.7)	145 (58.5)	74 (29.8)	
Troubles a lot	50 (6.5)	3 (6.0)	14 (28.0)	33 (66.0)	
Problems with psychosocial work conditions, n (%)					<0.001^a^
Not present/no harm/cannot say	415 (54.1)	176 (42.4)	193 (46.5)	46 (11.1)	
Troubles a little	263 (34.3)	45 (17.1)	150 (57.0)	68 (25.9)	
Troubles a lot	89 (11.6)	9 (10.1)	36 (40.4)	44 (49.4)	
Job demands, points (mean ± SD)	15.5 ± 4.0	14.1±3.6	15.5±3.8	17.4±4.3	<0.001^b^
Depressive symptoms, points (mean ± SD)	2.6 ± 0.9	2.2±0.5	2.6±0.8	3.3±0.9	<0.001^b^
Work ability, points (mean ± SD)	8.1 ± 1.7	8.8±1.3	8.2±1.5	6.8±2.1	<0.001^b^
General stress, n (%)					<0.001^a^
No	61 (8.0)	38 (62.3)	20 (32.8)	3 (4.9)	
Little	221 (28.8)	76 (34.4)	121 (54.8)	24 (10.9)	
Somewhat	322 (42.0)	92 (28.6)	168 (52.2)	62 (19.3)	
Quite a lot	127 (16.6)	20 (15.7)	57 (44.9)	50 (39.4)	
Very much	36 (4.7)	4 (11.1)	13 (36.1)	19 (52.8)	
HbA1c level^c^, n (%)					<0.001^a^
≤ 60 mmol/mol (≤7.5 %)	255 (33.2)	90 (35.3)	131 (51.4)	34 (13.3)	
61–70 mmol/mol (7.6 %–8.5 %)	271 (35.3)	85 (31.4)	135 (49.8)	51 (18.8)	
71–80 mmol/mol (8.6 %–9.5 %)	176 (22.9)	43 (24.4)	86 (48.9)	47 (26.7)	
≥ 81 mmol/mol (≥ 9.6 %)	65 (8.5)	12 (18.5)	27 (41.5)	26 (40.0)	
High blood glucose level at work, n (%)					<0.001^a^
Never	148 (19.3)	94 (63.5)	42 (28.4)	12 (8.1)	
Quite seldom	208 (27.1)	68 (32.7)	120 (57.7)	20 (9.6)	
Sometimes	226 (29.5)	45 (19.9)	133 (58.8)	48 (21.2)	
Quite often	150 (19.6)	20 (13.3)	74 (49.3)	56 (37.3)	
Always	35 (4.6)	3 (1.3)	10 (28.6)	22 (62.9)	
Difficulty in accepting Type 1 diabetes, n (%)					<0.001^a^
No	568 (74.1)	211 (37.1)	271 (47.7)	86 (15.1)	
Yes, a little	160 (20.9)	16 (10.0)	96 (60.0)	48 (30.0)	
Yes, a lot	39 (5.1)	3 (7.7)	12 (30.8)	24 (61.5)	
Severe hypoglycemia events in 12 months prior to enrollment, n (%)					0.023^a^
No	593 (77.3)	183 (30.9)	300 (50.6)	110 (18.5)	
Once	89 (11.6)	26 (29.2)	41 (46.1)	22 (24.7)	
2–3 times	53 (6.9)	17 (32.1)	24 (45.3)	12 (22.6)	
More often	32 (4.2)	4 (12.5)	14 (43.8)	14 (43.8)	

^a^Chi-square test

^b^ANOVA analysis

^c^Self-reported

**Table 2:** Correlation table for variables used in the structural equation modeling

	2	3	4	5	6	7	8	9	10	11	12	13	14
1 Gender	,076*	-,019	-,085*	-,002	-,172**	-,052	-,142**	-,036	-,124**	-,155**	-,224**	-,132**	-,097**
2 Age		,178**	-,096**	-,260**	,101**	,151**	,029	,061	,055	,158**	-,139**	-,054	-,003
3 Educational level			,035	,073*	,093**	-,083*	-,008	,082*	,077*	-,064	-,136**	-,074*	-,027
4 Duration of diabetes				,067	,029	-,043	-,113**	-,023	-,002	-,038	,146**	-,023	,003
5 Work ability					-,365**	-,396**	-,189**	-,184**	-,393**	-,433**	-,107**	-,229**	-,432**
6 Problems with psychosocial work conditions						,417**	,219**	,314**	,402**	,387**	,101**	,222**	,394**
7 Problems with physical work conditions							,151**	,310**	,314**	,464**	,071*	,290**	,307**
8 Difficulty in accepting Type 1 diabetes								,132**	,202**	,328**	,182**	,219**	,278**
9 Job demands									,332**	,310**	,067	,191**	,199**
10 General stress										,342**	,124**	,228**	,465**
11 Work-related diabetes distress											,180**	,393**	,434**
12 HbA1c level												,333**	,202**
13 High blood glucose level at work													,274**
14 Depressive symptoms													

*P Correlation is significant at the level 0.05 (two-tailed). **P Correlation is significant at the level 0.01 (two-tailed)

**Fig. 1:**
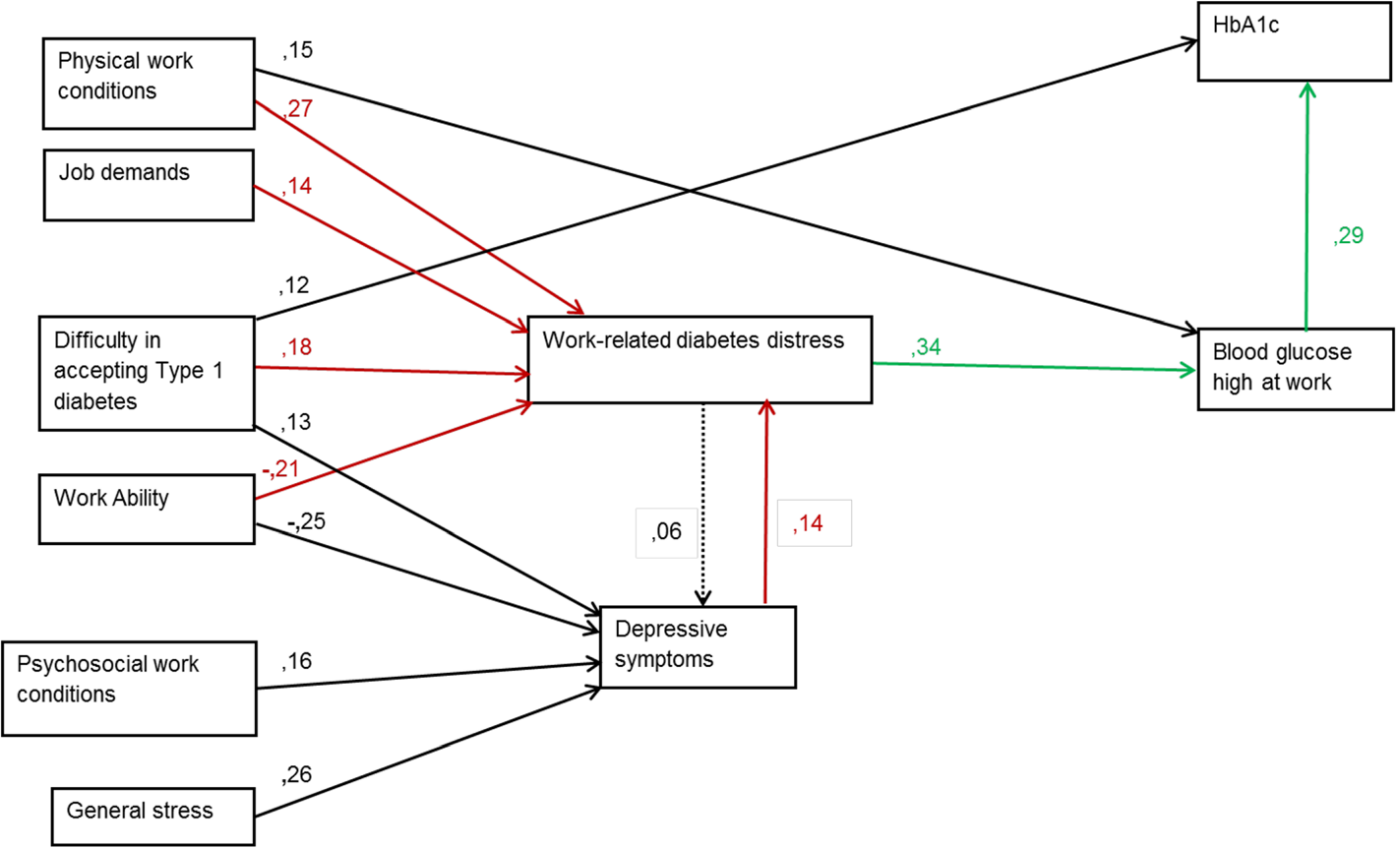
Path diagram of work-related, diabetes-related, and health-related variables with work-related diabetes distress. All of the path coefficients were standardized (β). This model was adjusted for gender, age, education level, and duration of diabetes. Gender was associated with HbA1c level (-,16) and work-related diabetes distress (-,10). Age was associated with a high blood glucose level at work (-,13) and depressive symptoms (-,11). Level of education was associated with the HbA1c level (-,12). Duration of diabetes was also associated with the HbA1c level (,16). Red arrows show the predictors of work-related diabetes distress. Green arrows indicate how work-related diabetes distress mediates the effect of these predictors on metabolic control.

We used structural equation modeling (SEM) [42] for analyzing the correlational multivariate data simultaneously. Basically, SEM conjoins the methodology of multiple regression and path analysis, in which the hypothetical causal relations are defined on the basis of previous research and theory. We used this method to clarify the association between the independent variables (physical and psychosocial work conditions, job demands, self-rated work ability, difficulty in accepting diabetes, and general stress) and the dependent variables (work-related diabetes distress, HbA1c level, high blood glucose level at work, and depressive symptoms). These potential and theoretically relevant variables were placed in the model according to previous studies [1, 9, 22, 26–31, 34–38, 41]. Standardized (β) and non-standardized (B) regression coefficients were computed [see Additional file 1], and a path diagram was drawn (Fig. 1). Standardized regression coefficients (β) were interpreted as weak if <0.10, moderate if 0.10–0.30, and strong if >0.50 [42]. Model fit was estimated with the GFI (goodness of fit index) and the RMSEA (root mean square error of approximation). A GFI (0–1) of >0.90 indicated a good model, and >0.95 indicated an excellent model. The RMSEA should be <0.08, and a value of <0.05 was considered excellent [43].

All of the analyses were performed with SPSS for Windows, Rel. 19.0.0.2. 2010 (SPSS Inc., Chicago, IL, USA) and IBM SPSS®Amos 21.0.0.

## Results

The descriptive statistics of the 767 participants are presented in Table 1. The mean age of the participants was 36 (SD 12.4, range 18–64) years, 56 % were men, and 62 % were married or cohabiting. The majority (91 %) worked as wage-earners, and over half worked in physical tasks (at least 50 % of their working time). The mean of the sum index for job demands was 16 (SD 4.0, range 5–25), that for depressive symptoms was 2.6 (SD 0.9, range 2–4), and the mean for self-rated work ability was 8.1 (SD 1.7, range 0–10). The mean duration of diabetes was 8.5 (SD 4.8, range 0–38 ) years, and 23 % declared that they had had one or more severe hypoglycemic events in the last 12 months.

Of the respondents, 30 % had never experienced work-related diabetes distress, 49 % reported having work-related diabetes distress sometimes, and 21 % reported such stress often (Table 1). The higher the HbA1c level, the more common work-related diabetes distress was. Of the respondents with good metabolic control, 13 % reported that they were often stressed. In contrast, among those with poor metabolic control, 40 % were often stressed. Of those who had experienced more than 3 severe hypoglycemia events in the past 12 months, 44 % reported often experiencing stress, whereas 19 % of those without such events had often been stressed. Altogether 63 % of those who always kept their blood glucose level higher at work than usual reported often having work-related diabetes distress.

Among the respondents with the highest education, work-related diabetes distress was reported markedly less often (Table 1). Those who perceived problems with physical or psychosocial work conditions experienced diabetes-related stress more often than the others. In addition, work-related diabetes distress was more common among those with much difficulty accepting their diabetes (62 %).

The correlations between the background, independent, and dependent variables are presented in Table 2. Work ability, problems with physical or psychosocial work conditions, difficulty in accepting diabetes, job demands, and general stress had the highest correlation with the dependent variables.

Figure 1 presents the path diagram of the structural equation modeling, which was adjusted for gender, age, educational level, and duration of diabetes. The regressions lower than 0.10 were removed from the final path diagram.

Problems with physical work conditions (β = 0.27), self-rated work ability (β = -0.21), difficulty in accepting diabetes (β = 0.18), and job demands (β = 0.14) were moderately associated with work-related diabetes distress. Work-related diabetes distress was moderately associated with the blood glucose level being maintained at a high level at work (β = 0.34). In turn, a high blood glucose level at work was associated with high HbA1c level (β = 0.29). In addition, physical work conditions were associated with keeping blood glucose high at work.

There was no notable association between general stress and work-related diabetes distress in the multivariate analysis. General stress (β = 0.26), self-rated work ability (β = -0.25), problems with psychosocial work conditions (β = 0.16), and difficulty in accepting diabetes (β = 0.13) were associated with depressive symptoms. Work-related diabetes distress and depressive symptoms showed a bi-directional association: diabetes distress was associated with depressive symptoms (β = 0.06) and depressive symptoms were associated with diabetes distress (β = 0.14) (Fig. 1). Separately, the associations were 0.19 for both directions.

Of the independent variables, only difficulty in accepting type 1 diabetes was associated with the three dependent variables: work-related diabetes distress, depressive symptoms, and HbA1c level.

The structural equation model was an excellent fit for the data, the GFI being 0.982 and the RMSEA being 0.039.

## Discussion

Working people with type 1 diabetes have work stress, just as their healthy colleagues do. They can also have diabetes distress, including diabetes self-management, and, in addition, work-related diabetes distress. We studied work-related diabetes distress in a representative national sample. The structural equation model enabled us to assess the theory-based associations of work-related, health-related, and diabetes-related factors with work-related diabetes distress.

In our study, work-related diabetes distress mediated the effect of independent variables on metabolic control. This finding is in a line with a previous study among people with diabetes [28].

We found an important association between problems with physical work conditions and work-related diabetes distress. The work environment and physical work load may be modifiable to better fit the needs of workers [44]; such an adjustment would probably decrease work-related diabetes distress. A high level of job demands, indicated by the pace and amount of work, was also associated with work-related diabetes distress. High job demands per se have previously been reported to be related to stress at work [9, 34]. It seems then that the same factors that increase stress in general also increase work-related diabetes distress. On the other hand, mental work and a high educational level seemed to protect workers from stress in our study. Therefore, persons with type 1 diabetes should be encouraged to use their full potential in pursuing their education.

Work ability showed a moderate association with work-related diabetes distress. We measured work ability using the Work Ability Score (WAS). This single-item score comes from the first question of the widely used Work Ability Index [40]. WAS has been found to be reliable and valid, and it is an easy method to assess work ability [22, 39]. Persons with type 1 diabetes and an excellent work ability probably have uncomplicated diabetes. Diabetes complications and other co-morbidities decrease work ability and induce work-related diabetes distress. In previous studies, work ability has been shown to correlate with productivity loss [45], as well as with mortality and disability [33]. A low WAS among workers with type 1 diabetes challenges occupational health personnel and employers to develop opportunities for part-time work and to modify jobs to match workers’ resources or arrange other means of support.

Difficulty in accepting diabetes was the only independent variable associated with the three dependent variables: work-related diabetes distress, depressive symptoms, and HbA1c level. These associations have been found also in an earlier study [41]. Learning to live with diabetes is a lifelong process; thus the duration of diabetes is not the “point” in how well a person has been empowered and has accepted his or her diabetes after receiving the diagnosis [46]. People develop and sustain various behavior patterns over time [47]. Although workplace health promotion may be one way to promote a change in health behavior [48], individual health care and support in self-management activities is needed to meet the different and varying needs of people diagnosed with diabetes [46, 47].

In previous studies, high diabetes distress was found to be correlated with poor diabetes empowerment, low overall and mental quality of life, low income, unhealthy diet, physical inactivity, poor glycemic control, hypoglycemic reactions, and future complications [3, 4, 38]. In addition, individual (e.g., knowledge and motivation) and environment-related (e.g., social support) issues affect the self-management of diabetes. Too, depression may cause poor diabetes self-care. Self-management education, coping skills, and the ability to solve problems have been found to decrease diabetes-related distress [38]. As a result, counseling, training, and coping courses for persons with diabetes are needed to support the self-management of diabetes.

An interesting result of our study was a lack of a notable association between general stress and work-related diabetes distress in the multivariate analysis. The latter seems therefore to be an independent phenomenon of general stress in the worklife of people with type 1 diabetes.

In this study, work-related diabetes distress was associated to the greatest degree with the blood glucose level being kept at a high level at work. If the physical work load is unpredictable, the risk of hypoglycemia increases. Furthermore, an inability or reluctance to self-monitor blood glucose at work may lead to the blood glucose level being kept high at work. Therefore a tendency to keep the blood glucose level high at work as a coping strategy may partly be due to a realistic fear of hypoglycemia and partly due to personal challenges in reaching good metabolic control. As far as we know, there are no reports concerning the problem of keeping blood glucose levels too high at work. The complications of poor metabolic control are well known, but the frequency and reasons for voluntarily maintaining a high blood glucose level are not. Therefore further studies are needed on this phenomenon.

Work-related diabetes distress had a bi-directional association with depression. Similar associations have also been found in other studies [37]. When both directions were estimated in the same model the association from depressive symptoms to work-related diabetes distress was stronger than vice versa. Nevertheless, as these directions are cross-sectional associations, we cannot make any causal inferences. It is reasonable to conclude that diabetes-related stress can increase the risk of depression [27, 28], and, obviously, depression-prone people also tend to feel stress about their work ability [49]. Screening for the risk of depression among workers with type 1 diabetes may facilitate early intervention aimed at improving work ability.

One of the strengths of our study was the size of the randomized sample (*n* = 2500), which covered 8.3 % of the working-aged Finns with type 1 diabetes. Even though only half of the recipients returned the questionnaire, the respondents seemed to form a good representation of working-aged Finns with type 1 diabetes since the sample was not biased in terms of demographic distributions. In addition, the sample size was big enough to enable a comparison of workers who had never experienced work-related diabetes distress with those who often felt such stress. Our sample represented workers extensively from different organizations and types of occupations. Furthermore, the questionnaire was based on several validated scales that included questions related to health, work ability, and worklife.

Using SEM, we were able to assess the associations, and the paths were dictated on a theoretical basis. Due to the cross-sectional nature of the study, the causal relations between the variables could not be confirmed, however. As the study was based on self-reporting, biases especially in the recall of the HbA1c level and the assessment of other study factors could have affected the results. Nonetheless, self-reported measurements are widely used due to practical reasons in data collection of large study samples (11, 29, 48). All of the participants were Finnish which may limit the generalizability of our results. We think that the results would be applicable to other Western countries, which have same kind of labour legislation and where health care is available for the whole population.

## Conclusions

In conclusion, work-related diabetes distress was found to be common among workers with type 1 diabetes. Problems with physical work conditions, work ability, difficulty in accepting diabetes, job demands, as well as depressive symptoms proved to be associated with work-related diabetes distress. Work-related diabetes distress seems to mediate the adverse effects of these factors on metabolic control through the maintenance of a high blood glucose level at work. Reconciling work with diabetes may be challenging. Some of the factors associated with work-related diabetes distress are modifiable, and some are not. Vocational guidance should encourage people with type 1 diabetes to use their full potential in pursuing their education. Occupational health personnel, employers, and superiors at work can facilitate and support the self-monitoring of blood glucose at work. They can also collaborate with people with diabetes to devise means for adapting work to fit their needs. We suggest that even minor work arrangements may be enough to diminish work-related diabetes distress.

## Additional file

Additional file 1: Table S1. The association of work-related, diabetes-related, and health-related variables with diabetes-related stress at work and HbA1c level, blood glucose high at work, and depressive symptoms. Standardized (β) and non-standardized (B) regression coefficients. (DOCX 14 kb)

## Abbreviations

ANOVA: analysis of variance
GFI: goodness of fit index
HbA1c: glycosylated hemoglobin
RMSEA: root mean square error of estimation
SEM: structural equation modeling
WAI: Work Ability Index
WAS: Work Ability Score
