# Supplementary material for: Work-related diabetes distress among Finnish workers with type 1 diabetes: a national cross-sectional survey
Source: J Occup Med Toxicol. 2016 Mar 21;11:11. doi: 10.1186/s12995-016-0099-4 (PMC4802881; doi:10.1186/s12995-016-0099-4)
Supplement: Additional file 1: Table S1. — The association of work-related, diabetes-related, and health-related variables with diabetes-related stress at work and HbA1c level, blood glucose high at work, and depressive symptoms. Standardized (β) and non-standardized (B) regression coefficients. (DOCX 14 kb) [file 12995_2016_99_MOESM1_ESM.docx]

|  | Work-related diabetes distress | | HbA1c level | | Blood glucose high at work | | Depressive symptoms | |
| --- | --- | --- | --- | --- | --- | --- | --- | --- |
|  | β-coefficient | B-coefficient | β-coefficient | B-coefficient | β-coefficient | B-coefficient | β-coefficient | B-coefficient |
| Gender | -0.099 | -0.253 ^<0,001^ | -0.161 | -0.304 ^<0,001^ |  |  |  |  |
| Age |  |  |  |  | -0.131 | -0.012 ^<0,001^ | -.112 | -.008 ^<0,001^ |
| Educational level |  |  | -0.124 | -0.119 ^<0,001^ |  |  |  |  |
| Duration of diabetes |  |  | 0.157 | 0.031 ^<0,001^ |  |  |  |  |
| Work ability | -0.208 | -0.157 ^<0,001^ |  |  |  |  | -.246 | -.124 ^<0,001^ |
| Problems with psychosocial work conditions |  |  |  |  |  |  | .157 | .093 ^<0,001^ |

(continued)

Table S1. Continued

| Problems with physical work conditions | 0.265 | 0.549 ^<0,001^ |  |  | 0.149 | 0.277 ^<0,001^ |  |  |
| --- | --- | --- | --- | --- | --- | --- | --- | --- |
| Difficulty in accepting Type 1 diabetes | 0.179 | 0.404 ^<0,001^ | 0.115 | 0.192 ^<0,001^ |  |  | .125 | .190 ^<0,001^ |
| Job demands | 0.136 | 0.043 ^<0,001^ |  |  |  |  |  |  |
| General stress |  |  |  |  |  |  | .264 | .233 ^<0,001^ |
| Work-related diabetes distress |  |  |  |  | 0.340 | 0.305 ^<0,001^ | 0.062 | 0.041 ^.376^ |
| Blood glucose high at work |  |  | 0.285 | 0.235 ^<0,001^ |  |  |  |  |
| Depressive symptoms | .140 | .209 ^0,035^ |  |  |  |  |  |  |
